# Service Coordination in Early Childhood Home Visiting: a Multiple-Case Study

**DOI:** 10.1007/s11121-023-01558-6

**Published:** 2023-06-27

**Authors:** Leeya Correll, Allison West, Anne K. Duggan, Kelsey Gruss, Cynthia S. Minkovitz

**Affiliations:** Department of Population, Family and Reproductive Health, Johns Hopkins Bloomberg School of Public Health, Baltimore, USA

**Keywords:** Home visiting, Service coordination, Early childhood, Multiple-case study, Qualitative

## Abstract

Early childhood home visiting is a preventive service delivery strategy that aims to promote child and parent health, positive parenting, child development and school readiness, and family economic self-sufficiency. To meet families’ needs, programs provide a combination of direct services, and referrals and linkages to community-based services. Service coordination is therefore a critical component of home visiting’s role within the early childhood system of care. This multiple-case study describes facilitators and barriers to service coordination, as identified by home visiting program staff and families. We interviewed program managers, supervisors, home visitors, and families from four local home visiting programs in the eastern USA with diverse contextual characteristics that showed evidence of having strong coordination. Using multiple-case study methodology, we analyzed the data to understand key facilitators and barriers to service coordination for each case and identify and describe common themes across cases. Facilitators included interagency relationships and collaboration; a culture of teamwork; dedicated, well-connected staff; supervision; trusting relationships with families; and warm handoffs. Barriers to service coordination were limited availability and accessibility of local resources, perceived stigma among other service providers, and families’ ambivalence toward some services. Home visiting staff and families emphasized that relationships at multiple levels are fundamental to service coordination, barriers are complex, and coordination is time- and labor-intensive. Coalitions that bring together diverse stakeholder groups at the state and local levels can provide meaningful coordination support to the early childhood services.

**Supplementary Information:**

The online version contains supplementary material available at 10.1007/s11121-023-01558-6.

## Introduction

Maternal and early childhood home visiting is a preventive service strategy that provides voluntary home–based services to expectant families and families with young children (Minkovitz et al., 2016). The primary objectives of home visiting are to improve child outcomes and support parenting during critical periods of family health and child development. Home visitors serve provide a combination of direct services, such as parenting education and support, and referrals and linkages to other community services (USDHHS, 2021). Most home visiting programs aim to serve families with multiple, complex challenges, including economic vulnerability, child health or developmental challenges, mental health or substance use concerns, partner violence, and child maltreatment (Adirim & Supplee, 2013; Duggan et al., 2018).

Evidence-based home visiting services have been scaling up in the USA since 2010, when the Affordable Care Act established the federal Maternal, Infant and Early Childhood Home Visiting (MIECHV) Program and invested $1.5 billion towards implementing evidence-based home visiting program models (Adirim & Supplee, 2013). To date, 22 models have met criteria for evidence of effectiveness and several other models are currently being evaluated (USDHHS, 2021). In FY 2020, MIECHV-supported home visiting served 140,000 parents in 50 states, the District of Columbia, and five territories (USDHHS, 2021). In many communities, home visiting services are supported by non-MIECHV funding, including additional federal, state, and local public and private initiatives.

Despite the large investment in home visiting, many impact evaluations report modest effect sizes (Supplee & Duggan, 2019). For example, a randomized clinical trial of Nurse Family Partnership found no effects on birth outcomes (McConnell et al., 2022). Nurse Family Partnership and other evidence-based home visiting models rely on home visitors providing families with referrals and linkages to other service providers to address some identified needs; thus, mixed and null effects on intended outcomes may be attributable, at least in part, to challenges with service coordination (Moniz et al., 2022).

*Service coordination* refers to the “organization of activities between two or more organizations to facilitate, in partnership with the family, the delivery of the right services in the right setting at the right time” (West et al., 2018a). Service coordination has many potential benefits for families, home visiting programs, and other early childhood services. Service coordination has been associated with increased retention in home visiting services, increased cessation of prenatal smoking, and decreased emergency department use for childhood injury (Williams et al., 2020). Collaborative efforts to improve service coordination for maternal depression in home visiting have resulted in improved access to services and improved depression symptoms (Tandon et al., 2020). Other potential benefits of coordination include improved relationships between home visiting staff and other community providers, facilitation of referrals between providers, mutual reinforcement of anticipatory guidance, and increased timeliness and efficiency of services received (Sides & Baggett, 2015; West al., 2018a). In the long term, these benefits may contribute to enhanced child and family health and well-being (Tandon et al., 2020; Toomey & Cheng, 2013; Tschudy et al., 2013; Williams et al., 2020).

In home visiting, service coordination is a complex process with multiple steps, including screening, referral, linkage, and follow-up (West et al., 2018a). Each step requires one or more activities on the part of the referring home visitor, the family, or the organization to which the family is being referred. Screening identifies needs for services beyond what the home visiting program can provide. A referral provides key information about a service and service provider to the family. Linkage connects the family to the provider and may include sharing information to the provider about the family (with the family’s permission). Finally, follow-up closes the loop with the family and provider to ensure that a high-quality service was received.

Recent evidence highlights the complexity of the process and potential challenges in implementation. For example, a mixed methods study of five home visiting sites in Massachusetts found that only 21% of referrals resulted in connections to services (Goldberg et al., 2018). The same study documented variation in the home visitor effort required to connect participants to services. Considerably more effort was required for services for which local resources were scarce, such as housing. A national survey of home visiting sites revealed variability in coordination supports; on average, sites had stronger systems in place to support screening and referring families than to support linkage and follow-up (West et al., 2021). Moreover, sites evidenced stronger supports for coordinating services for mental health and partner violence than for substance use. Finally, there is evidence of marked variability in how sites collect and track screening, referral, linkage, and follow-up data, which may be influenced by family, provider, and community characteristics (Rosinsky et al., 2019).

To address the need for further research on service coordination in home visiting, we previously used an evidence- and expert-informed process to develop The Measurement Framework for Coordination (West et al., 2018a). The Framework specified 37 indicators to assess the strength of the implementation system and activities that support successful service coordination between home visiting and other services within the early childhood system. Separate manuscripts have described the development of the framework (West et al., 2018a) and reported results of quantitative studies of service coordination in home visiting (West et al., 2020, 2021). An overview of the framework and its specific inputs, activities, and outcomes is available as supplemental content.

This qualitative multiple-case study aims to add to the larger service coordination study by enriching and expanding our understanding of factors contributing to and impeding coordination. Specifically, researchers, program implementers, and policymakers need a better understanding of how state and local contexts, organizational characteristics, and family characteristics influence service coordination. The perspectives of local home visiting staff and families enrolled in home visiting should inform this understanding. Using qualitative multiple-case study methodology, the aim of this study was to develop a deeper understanding of factors that support service coordination. To accomplish this, we sampled four home visiting sites from the eastern USA that showed evidence of having strong systems to support coordination in an earlier, quantitative phase of the study.

## Methods

### Study Design

Following Yin (2014), we used a multiple-case study design to examine *how* four high-performing home visiting programs support service coordination in their work with other child and family-serving agencies and organizations. Case studies are useful for in-depth study of individuals’ perceptions and perspectives of complex factors operating within and outside the organizational context. Multiple-case study methodology allows for exploring commonalities and differences between sites and leveraging and triangulating varied data sources such as staff interviews, client interviews, field notes, and relevant documents (Marshall & Rossman, 2014). We defined a “case” as one home visiting program.

We also aligned this study with a positive deviance approach, which presumes that “knowledge about ‘what works’ is available in organizations that demonstrate high performance in an area of interest” (Bradley et al., 2009). Following this approach, we selected four sites that showed evidence of having strong systems in place to support coordination. Knowledge generated from these high-performing sites contains lessons that can be applied to sites that have relatively weaker service coordination systems and activities.

### Sample and Recruitment

A multi-step stratified purposeful sampling strategy facilitated comparison and theoretical replication to improve our understanding of how and why programs across various dimensions of heterogeneity successfully coordinated services (Yin, 2014). We selected the four cases from 89 home visiting sites from across the USA that participated in a survey on service coordination (West et al., 2020). Sites were members of a large practice-based research network of the Home Visiting Applied Research Collaborative and varied in program model, geographic context, size, and receipt of federal MIECHV funds (O’Neill, 2018). Survey questions assessed hypothesized indicators of coordination that were developed in an earlier phase of the project (West et al., 2018a).

We used a three-step process to identify potential cases for inclusion. First, we used the survey data to create a score reflecting the strength of each site’s implementation system and activities to support service coordination, based on the conceptual framework. Implementation system indicator items related to programmatic supports for four key aspects of coordination (screening, referral, linkage, and follow-up), for each of four service needs (mental health, substance use, intimate partner violence, and child developmental delay). Further details on the survey and summary score calculations are described elsewhere (West et al., 2021). We then identified the top third of all sites based on these scores. Finally, we used a maximal variation approach (Patton, 2014) to select sites that demonstrated variation on key contextual characteristics, including home visiting program model, program size, geographic location, receipt of MIECHV funding, and whether programs served urban, suburban, and/or rural communities. We approached five sites and four agreed to participate.

From each site, we aimed to recruit a program manager (PM), a supervisor (SV) when available (for two sites, the same person served as both the PM and SV), two home visitors (HVs), and four families to participate in interviews. To be eligible to participate, PMs must have worked at the site for 2 years, SVs for 1 year, and HVs for 6 months. The study team asked each participating HV to refer two families from their caseload to participate in an interview and assisted HVs with randomly selecting eligible families. Families were eligible if the primary caregiver was at least 18 years old, spoke English, had been enrolled in home visiting for at least 6 months, and had been referred for at least one service, such as for counseling, child development services, or substance use treatment.

### Data Collection

Between October 2017 and January 2018, two study team members conducted site visits at the four programs. They completed staff interviews in-person, collected documents, and toured each site. One study team member conducted the family interviews by phone within 2 weeks of the site visit. Interviews were audio recorded, de-identified, and transcribed verbatim.

Previous home visiting service coordination literature and the Measurement Framework for Coordination informed the interview guide. The study team piloted and refined the HV, PM, and family interview guides based on participant feedback. The team also tailored the guides to the interview participants’ roles. Staff interviews were designed to take about an hour to complete and included open-ended questions about the local and organizational context, site implementation systems and activities related to service coordination, and perceived facilitators and barriers to achieving successful service coordination. We asked home visiting staff to provide their definition of service coordination, describe how it works in their program, and describe their programs’ expectations for coordinating services with families around concerns such as mental health and substance use, child developmental delays, and intimate partner violence. Family interviews were designed to take about 30 min to complete and asked open-ended questions about families’ perceptions of and satisfaction with service coordination processes, including the referrals that they received and how their home visitor made the referral, linked them with services, and followed up with them about the referral. We conducted 30 interviews total, with two PMs, two PM/SVs, two SVs, eight HVs, and 16 family members.

We also collected and reviewed programmatic documents. We asked PMs to provide copies of any documents related to service coordination, including: forms to guide intake, screening, referral, linkage, and follow up; job descriptions; policies and procedures on service coordination; forms used in supervision; data sharing agreements; and any other policies, procedures, or forms used to guide, support, or track service coordination.

### Data Analysis

The study team analyzed the interview data using Dedoose (2018). To develop the coding scheme, we used an integrated approach that employed both a deductive organizing framework and inductive development of codes (Bradley, 2007). Investigators first developed a list of codes based on the Measurement Framework for Coordination (West et al., 2018a). The Framework details key domains and indicators for coordination of services between home visiting and other child- and family-serving programs. The first and second authors independently reviewed two interview transcripts and applied the list, adding codes as necessary to capture new phenomena. Using an iterative process, the coding team revised codes and operational definitions as needed. The final codebook was applied to all interview transcripts. At least two team members coded each interview transcript. Discrepancies between coders were resolved through in-person meetings and discussion. The study team reviewed programmatic documents for contextual information and to triangulate findings from the other data sources.

As a first step of synthesis, investigators drafted an individual case study for each program using a common template. To promote credibility, we conducted member checks by sharing drafts with staff at each site to verify whether the results resonated with their experiences (Birt et al., 2016). Investigators incorporated feedback from sites in the final individual case study drafts. The first author then conducted a cross-case synthesis to compare themes across cases. Following Yin (2014), she arrayed the data from the four cases in tables. Themes were defined as patterns within the codes, and the themes presented in this report are those that were common across two or more sites. The first and second authors examined themes regarding the direction of influence (facilitator or barrier to service coordination) in each case and reviewed and interpreted results during regular debriefings.

## Results

Table 1 presents key characteristics of each case. The four cases represented three home visiting models and were located in the Northeast, Southeast, Appalachia, and Mid-Atlantic regions of the USA. Programs ranged from 40 to 130 families served. Non-profit organizations implemented all programs. They had a variety of co-located services, such as Early Head Start and food banks. One program (Case 3) did not receive federal MIECHV funding.

**Table 1:** Context for four case study sites

	Case 1	Case 2	Case 3	Case 4
*Geographic context*				
Community type	Rural	Urban, suburban, and rural	Suburban	Suburban
Region	Northeast	Southeast	Appalachia	Mid-Atlantic
*Organizational context*				
Home visiting program model	Parents as teachers	Healthy Families America	Maternal Infant Health Outreach Worker Program	Parents as teachers
Number of families served	60	130	40	50
Implementing agency	Non-profit	Non-profit	Non-profit	Non-profit that serves as a central point of entry for other early childhood services
Co-located services	Food bank, diaper pantry	Home visiting program for grandparent caregivers; program offering hospital visits to all new parents in the county; food, clothing, and diaper resources	Early Head Start	Early Intervention and Early Head Start, on-site access to mental health consultant, nurse, case management services for families with intensive needs
MIECHV^a^ funding	Yes	Yes	No	Yes
Data system	MIS^b^ developed by state agency	MIS developed by program model	MIS developed by state agency; state in process of switching systems, currently using paper	Introducing new MIS developed by program model

^a^*MIECHV* The federal Maternal, Infant, and Early Childhood Home Visiting program

^b^*MIS* Management Information System

In examining facilitators and barriers to service coordination across cases, we identified several themes. We organized the themes under five key domains of coordination drawn from the Measurement Framework for Coordination: state and local context, organizational context, implementation system, family factors, and activities. Table 2 indicates which cases identified the themes as facilitators and barriers to coordination. Table 3 presents exemplar quotes.

**Table 2:** Facilitators and barriers to service coordination across cases^a^

	**Case**			
**Theme**	1	2	3	4
State and local context				
Interagency relationships and collaboration	↑	–	↑	↑
Limited availability and accessibility of local resources	↓	↓	↓	↓
Stigma from other service providers	–	–	↓	↓
Organizational context				
Culture of teamwork	↑	↑	–	–
Dedicated, well-connected staff	↑	↑	↑	↑
Interpersonal relationships with other early childhood organization staff	↑	–	↑	↑
Implementation system				
Supervision	↑	↑	↑	↑
Program strategies for compiling and disseminating information on community resources	↑	↑	↑	↑
Family factors				
Ambivalence	↓	↓	–	↓
Activities				
Trusting relationships with families	↑	–	↑	↑
Family empowerment	–	↑	–	↑
Tailored approach	↑	–	↑	↑
Warm handoff	–	–	↑	↑

↑indicates facilitator to service coordination, ↓ indicates barrier to service coordination, and – indicates no mention or neutral

^a^For representative quotes, see Table 3

**Table 3:** Themes and representative quotes

**Theme**	**Quotes**			
State and local context				
Interagency relationships and collaboration	“[The state coalition is] a wonderful group of people, not just because we get together and have trainings, but we get together and we talk about what are our barriers…And because we’re spread across the whole state…it’s a great time to talk about what are ways that you’ve overcome those barriers, and maybe I could do that in my area, too.” [Case 3 PM/SV]			
Limited availability, accessibility, and quality of local resources	“I think our geographic, where we are. There is a limit of services. You have one mental health provider that is a sliding scale fee. Anything else is going to be private…. They may go to [mental health services] once or twice. It’s intimidating, first of all…. Then, they get in there, and there’s a long wait. Oftentimes, they’ll get up and leave” [Case 2 PM]			
Stigma from other service providers	“The [Hispanic] parents tell you, ‘Oh, but they didn’t treat… the American that way. They left me outside the hallway there, while the American, they let them in.’” [Case 4 HV]			
Organizational context				
Culture of teamwork	“One of us could bring a case to the table and say, ‘Well I’m having this problem with [family]. Has anyone got suggestions to how we can do it?’ Then we start talking about it…. In a difficult situation everybody comes in. Brainstorms…. We’ll say, ‘Well we need to get together and get this done.’” [Case 2 HV]			
Dedicated, well-connected staff	“If you don’t have the right people on the job…you get a parent educator that’s not really in it for the right reason. You’re not gonna see the same success. And since the salaries are so low, my parent educators make what they make at Walmart…they have to really be dedicated to do what they do.” [Case 1 PM/SV]			
Interpersonal relationships with other early childhood organization staff	“If there’s a committee, we want to be a part of it. It's that relationship building, again. If you don’t have relationships with [other service providers] and they don’t know who you are they’re less likely to help you.” [Case 3 HV]			
Implementation system				
Supervision	“When you’re running from one visit to the next visit, it’s very easy to go, ‘Oh, I did talk with the family and I forgot to give them this information or I didn't bring it,’ and then a referral’s not actually made. So [reflective supervision time] is a very regular check in about the families…and then just organically it comes up, ‘Oh yeah, I made this referral,’ or ‘Maybe I should make that referral.’ And then ‘Hey, how'd that go?’” [Case 4 SV]			
Program strategies for compiling and disseminating information on community resources	“The Family Resource Network for this county puts together a lovely brochure and lists all the different social service agencies and what group would they fall under. Is it early childhood? Is it food or utility? That kind of thing. We make sure that families get those as they’re refreshed. We carry them on us. It’s a good way to leave it, and say, ‘Here you go. Here's a whole list of places that do food. Hang this on your fridge and keep it.’” [Case 3 PM/SV]			
Family factors				
Ambivalence	“Biggest barriers I would say would be probably follow through with the families…. The family may not see a need in the way that the home visitor sees it or they may not see it at that time. Sometimes the buy-in is not there.” [Case 4 PM]			
Activities				
Trusting relationships with families	“I just love the fact that that support system is there. And they are very trustworthy – anything you tell them, you don’t have to worry about such and such knowing about it…it’s just between us two.” [Case 4 Family]			
Family empowerment	“Giving them options. Asking the family what they’ve done before that’s worked. Has this ever happened before? What did you do? What kind of support systems you already have in place? We really encourage home visitors to not take that on but to make sure that they are acknowledging the family already has a support system if they have support systems…to think about how they might overcome…so that they are empowered to know how to help themselves.” [Case 4 PM]			
Tailored approach	“It’s going to be based on family needs and what their expectations are. If they’re not looking for medication assisted treatment, then I'm not going to take them there…. It probably depends on what the service is, whether it's that warm hand off or a phone call. Sometimes the families might be interested but they’re not ready, and sometimes you don’t want to get in the middle.” [Case 3 HV]			
Warm handoff	“Once they introduced me to the service provider, it basically just took off from there. It became…everybody would talk to each other and then I would talk to the service provider. Any more things that they had, I would go.” [Case 1 Family]			

*HV* home visitor, *SV* supervisor, *PM* program manager

### State and Local Context

Cross-case synthesis revealed variation in how state and local context influenced coordination. We identified three key themes: interagency relationships and collaboration; availability, accessibility, and quality of local resources; and stigma from other service providers.

#### Interagency Relationships and Collaboration

Three programs participated in coalitions of early childhood service providers and stakeholders at the local county or state levels, and one program participated in both a local and state coalition. Each identified that participation in the coalitions facilitated service coordination; it promoted mutual awareness and understanding of organizations’ roles within the early childhood system, enabled communication between service providers, and led to productive discussions regarding community needs, gaps in services, and areas to improve service capacity. An SV from Case 2 described how participation in a local coalition facilitated service coordination: “Most of the service providers locally are represented every month. We'll talk about what's happened in the community. What is your organization doing? Are there needs that your organization has that my organization might be able to fill?”.

#### Limited Availability, Accessibility, and Quality of Local Services

Limited availability, accessibility, and quality of local services were commonly identified barriers to service coordination. Specifically, Cases 1, 2, and 3 emphasized limited availability of mental health and substance use services in their communities. Case 4 staff expressed that families had difficulty affording the higher quality services available in their community. All four cases identified transportation as a barrier to service coordination. Staff described various strategies to address transportation barriers including providing transportation directly to families and assisting families with navigating the public transportation system.

#### Stigma from Other Service Providers

Cases 3 and 4 identified perceived stigma toward families from other service providers as a barrier to coordination. Some home visiting staff perceived other providers as uninterested or unwilling to prioritize working with their families. As the PM/SV from Case 3 described,

In our area, there’s a lot of agencies who don’t see the benefits of partnering with home visitation. It’s for ‘those people’. We don’t see ‘those people’…. ‘Those people’ is kind of a generic term that’s always used for the stereotypical poor person.

An HV from Case 4 reported that Hispanic families described poor treatment by other local service providers compared to non-Hispanic clients.

### Organizational Context

We identified three organizational context themes: culture of teamwork; dedicated, well-connected staff; and interpersonal relationships with other early childhood organization staff.

#### Culture of Teamwork

Staff at Cases 1 and 2 emphasized the importance of communicating with coworkers about service coordination issues. An HV from Case 1 described how communicating with other HVs facilitated her work as, “If I have something going on in one family and somebody else has had that same thing, to just be able to communicate and see how they handled something.” HVs cited brainstorming with their colleagues to learn from their collective experiences working with families and service providers.

#### Dedicated, Well-Connected Staff

Staff characteristics facilitated coordination across all four cases. These characteristics included dedication to the job, being invested in serving the community, and knowledge of and personal connections with local resources. An SV from Case 4 stated,

Some [staff] have been here a long time and they know a lot of resources and can make a personal connection…. [HV] knows everyone in the community, he’s been around a long time, and he can tell you who what when where…he knows it all.

The PM from Case 2 emphasized that all the HVs were from the community that they served, stating, “This is their community, and they're really invested in these moms getting what they need.” The PM from Case 1 also described HVs as “dedicated.” The SV from Case 3 emphasized the importance of personal dedication; she described herself as “always in everyone’s face” and identified this as a reason for their success.

#### Interpersonal Relationships with Other Early Childhood Organization Staff

Three of the programs identified relationships with other early childhood organizations as a facilitator of service coordination. Case 1 staff described building interpersonal relationships with other organizations by attending their events and inviting other organizations to their events. Similarly, Case 4’s PM identified attending community events and serving as board members for other organizations as helpful for building relationships with other services, stating,

The partnership piece, making sure that those community partnerships are in place hopefully long before referral is needed. It really takes some work having those connections. If we have an event like the community baby shower, having those vendors present and getting to know each other and what the other program does really facilitates the referral process…. We’re able to say not just, ‘Oh, go call [pediatric clinic] but ‘Oh, you want to talk to Dr. So-and-so over there.’ Have more of a warm connection.

### Implementation System

Across cases, supervision of HVs and procedures for gathering and disseminating information on community resources were essential implementation supports for coordination.

#### Supervision

All four cases identified supervision as a key facilitator of service coordination. Supervision was described as an opportunity for HVs to discuss barriers and strategies for service coordination. Specific supervision strategies varied between cases. The SV and HVs from Case 1 described “open-door policy” supervision, where HVs were encouraged to go to their SV at any time to talk through specific challenges. The SV and HVs from Case 2 described how the SV used role-play with HVs to prepare for challenges they may encounter with families. The PM described the rationale behind this, stating, “When [the HVs] go out there, it's not the first time they've had that conversation.” Cases 2, 3, and 4 identified reflective supervision strategies as facilitators. The SV from Case 2 described reflective supervision as,

Growing the relationship between you and the home visitor, and the home visitor and the family. It’s a parallel kind of process. What I’m doing with her, I would like to see that being done in the community with their families. Talking about and assessing what’s happening for their families.

#### Program Strategies for Compiling and Disseminating Information on Community Resources

Each program relied on a specific strategy for compiling and disseminating information on community resources to which they might refer families. For example, Case 2 staff provided families with a Community Resource Book at their first visit. HVs reviewed the book with families to prompt discussion of needed services and to empower families to contact services themselves. One HV in this program gave an example of how resource lists can help guide conversations about family needs:

She may not share with me a lot of things, but once she looks in the referral book she’s like, ‘You know, my lights [are] going to be turned off next week. Oh, look, there’s someone I can call to see if they can help.

Case 4, which was housed within a large nonprofit and co-located with several other services, used a weekly email to share information about local resources and services with program staff.

### Family Factors

One theme related to family factors was identified: the perception that some families felt ambivalent about their need for services.

#### Ambivalence

Cases 1, 2, and 4 identified families’ ambivalence as a barrier to service coordination. As one HV stated, “The barrier sometimes [is] the mom just not doing something. That’s a choice once we refer her to it. We know she has transportation to get there, and she just opt not to do it.” Some HVs shared that at times they perceived a lack of agreement between what the family and the HV identified as a need and that this could lead to poor follow through. Some programs had strategies in place to help manage ambivalence. For example, a family member interviewed in Case 1 described her initial resistance to referrals for mental health services. In this situation, the HV brought her SV along to a subsequent visit, and together they were able to link the mother with counseling services successfully. The family member stated,

[The home visiting program] was so proactive. They helped me reach out to therapy to talk about what are the risks of the post-partum, understanding the signs and… if it wasn’t for [the home visiting program] being there for me and helping me get the linkage that I need, I wouldn’t have been able to do it.

### Activities

Cross-case synthesis revealed four common activities that supported coordination: establishing trusting relationships, developing a tailored approach for each family, facilitating warm handoffs, and family empowerment.

#### Trusting Relationships with Families

Three of the programs emphasized the importance of trusting relationships with families as an essential precursor to service coordination. As described by one HV, “I try to establish a relationship with them and let them know that they are important to me. ‘Cause I need that trust – it’s very important” (Case 3). The PM/SV from Case 3 described the first call to connect with a service as a potentially “scary moment” for families. She went on to describe how HVs can be more supportive and helpful in these situations when they have earned families’ trust: “And so that's a great opportunity is that phone call, while you are in the household with them, sitting beside your mom, and say, I'm here for you, and I'll help you through this scary moment.”

Families also described trust as important for successful service coordination. A family member from Case 4 described her HV as the “main one in my corner” when it came to helping her connect with mental health services. Another family member from Case 4 stated, “They are very trustworthy. Anything you tell them, you don't have to worry about such and such knowing about it, or he knows about it, it's just between us two.”

#### Family Empowerment

Staff from Cases 2 and 4 emphasized that they aimed to empower families through their approach to service coordination. HVs wanted families to build on their own resources and supports, with the hope that they would be able to sustain these connections after they left the program. As described by one HV from Case 2,

If I would have said, ‘Okay, I’ll be here at 10 to pick you up,’ then I’m starting something that is not helping her because I’m going to try to empower her to think and get these things done on her own.

The PM from Case 4 described how “doing with, not for” empowered families, when HVs “encourage the family to think about how they might overcome or how they might deal with [challenges] so that they are empowered to know how to help themselves.”

#### Tailored Approach

HVs from Cases 1, 3, and 4 tailored their approaches to service coordination according to the service needs and preferences of each family. An HV from Case 4 described her approach to tailoring as “meeting families where they are.” She stated,

When it comes to actually working with the families, I like getting into trenches and doing what’s going to help them. Maybe some programs say you have to do this, this, this, and you come out to the family and it’s really not where they’re at. I like to get to know each family, see where they’re at, and customize the fit just where they’re at.

An HV from Case 3 stated that she decided how much to assist families with referrals and linkages based on the family’s level of interest and readiness to be connected with services.

#### Warm Handoffs

HVs in Cases 3 and 4 described using warm handoffs, in which the HV connected families to providers directly, to facilitate service coordination. An HV from Case 3 described warm handoffs as encouraging another level of “accountability” for other service providers to follow up with families, because they knew that the HV would come back and check in to see how services were received. An HV from Case 4 stated that she accompanied and introduced families to new service providers for their first visit to improve their comfort level.

## Discussion

Home visiting is uniquely positioned to help families identify and obtain needed services in their communities. There is limited published research on service coordination in home visiting; the results of our multiple-case study add to this literature by illustrating the complexity of service coordination among home visiting and other early childhood services, as described by home visiting staff and families from sites that showed evidence of having strong systems in place to support service coordination, based on their responses to an earlier survey. Results suggest that successful coordination depends on sites having strong implementation supports, activities, and relationships across multiple levels of the early childhood system.

### Implementation Supports and Activities

A strong implementation system and activities for service coordination include infrastructure and key drivers such as policies and procedures, staff supervision, systems that support monitoring of referrals and outcomes, and specific strategies for working with families to support screening, referral, linkage, and follow-up (Fixsen et al., 2013; West et al., 2018a). All programs indicated the importance of maintaining a comprehensive, up-to-date list of available community services and resources to reference when referrals were needed. Moreover, programs that were co-located with other services were able to maintain up-to-date information on co-located services more readily. Co-location has been found to benefit home visiting programs by enabling administrative efficiencies and collaborative working relationships (Kellom et al., 2018); therefore, co-location may be a successful strategy for promoting service coordination and warrants further investigation.

Three programs specified the importance of reflective supervision, a model of supervision that is widely supported in home visiting and other early childhood services (Tomlin et al., 2014). Reflective supervision is considered essential to family-centered practice because it encourages HVs to consider their own feelings, beliefs, and biases about families and their circumstances (Bernstein & Edwards, 2012; Tomlin et al., 2009). Thus, reflective supervision offers an important opportunity for HVs to discuss barriers to service coordination, such as possible reasons for families’ ambivalence, and explore paths forward.

Two programs emphasized the importance of warm handoffs when linking families with services. Warm handoffs have been shown to be effective in improving linkages to services in certain settings, especially when services are stigmatized or are not easily accessible (Taylor & Minkovitz, 2021). More research is needed to determine the utility of warm handoffs in home visiting (Taylor & Minkovitz, 2021).

### Relationships at Multiple Levels Are Central to Service Coordination

Trusting relationships appear to undergird all aspects of coordination. Trusting relationships were important at multiple levels — between programs, within programs, and with families. All programs emphasized the importance of having staff who were well-connected with their communities. Participation in local and state coalitions increased awareness of local services and agencies and strengthened relationships between providers. Prior research has shown that interpersonal relationship quality is an important factor for successful collaboration (Mattessich et al., 2001). Our findings suggest that strong interpersonal relationships between staff at the referring and receiving agencies may also facilitate warm handoffs.

In addition to supervision support, HVs found coworker support to be invaluable to service coordination. These findings are consistent with a recent national study of home visiting staff, which found that HVs valued support, trust, and guidance from coworkers (Sandstrom et al., 2020). Finally, HVs and families across sites emphasized the critical role of a trusting HV-family relationship in service coordination. This is consistent with prior research, which has shown that positive helping relationships and HV “soft skills” are positively associated with engagement (Beasley et al., 2018; Bower et al., 2020; Korfmacher et al., 2007; Sandstrom et al., 2015) and parents’ willingness to disclose sensitive experiences (Jack et al., 2017).

### Barriers to Service Coordination Are Complex

Our multiple-case study identified several complex barriers to service coordination, such as limited availability and quality of local services, family ambivalence, perceived stigma, and time. It is clear that some referrals do not result in successful connection to services due to limited availability or low-quality services. Findings from this study are consistent with previous work that highlights concerns regarding the availability of high quality services, particularly for community-based mental health and substance use treatment services (Neger & Prinz, 2015). This is also consistent with findings from a recent qualitative analysis of home visiting service records, which found that successful linkages with services were contingent on the quality, capacity, and strength of the service systems to which they were referred (Goldberg et al., 2018).

There are many possible underlying reasons for what HVs perceived as families’ ambivalence towards referrals to services. Behavior change is known to be influenced by multiple factors (Glanz et al., 2008). For example, the transtheoretical model (TTM) of health behavior change posits that individuals progress through a series of six stages of change (Prochaska & Velicer, 1997). According to the TTM, a family may screen positive for a given risk (e.g., substance use), yet be in a stage of precontemplation in which they are unaware or unable to accept their problem and thus do not intend to act in the foreseeable future. HVs may identify families in a precontemplation stage as “not wanting to do it” or “in denial” (Table 3). HVs may be more successful in helping mothers resolve ambivalence if they can identify the family’s stage of change and tailor their communication accordingly.

Families may be hesitant to receive referrals to services for several additional reasons. For example, a HV may not fully explain the importance of a service in a way that the family understands. Other families may face more immediate priorities or crises that require their time and energy. Mental health challenges may also interfere with a family’s desire or ability to access a service. HVs in this study emphasized the need to tailor their approaches to service coordination depending on these and other family characteristics. This is consistent with recent efforts to promote precision home visiting, which focuses on identifying what works for whom and in what contexts and tailoring services accordingly (Supplee & Duggan, 2019).

Relatedly, we found that perceived stigma may be a barrier to service coordination. A growing body of literature indicates that stigmatizing attitudes and behaviors of health and social care professionals are important concerns for those who need mental health (Schulze, 2007; Wang et al., 2018) and substance use (Browne et al., 2016; van Boekel et al., 2013) treatment services. Provider stigma may negatively influence the quality of the helping relationship with the client, client satisfaction with services, and treatment outcomes (Charles, 2013). One case specifically identified provider stigma experienced by Hispanic families; this is consistent with previous research suggesting that language and cultural barriers between care providers and seekers are barriers to service utilization for Hispanic people (Rastogi et al., 2012).

Finally, findings from this study affirm that service coordination requires a great deal of time and labor on the part of home visiting programs. Home visiting staff noted that acquisition of local knowledge and relationships with early childhood service providers and families take time and effort to develop. Failure to consider the importance of these factors may underestimate the true time and effort needed for effective service coordination.

## Limitations

Although our multiple-case study contributes novel qualitative evidence to a vital and little studied aspect of home visiting service delivery, it has several limitations. First, specific case study findings may be unique to the selected programs. However, because we purposively selected programs to vary by geographic region, program model, and size, the themes and lessons learned are likely to be transferrable. We also incorporated field notes, memo writing, member checking, and data triangulation to strengthen the rigor and trustworthiness of our findings. Future qualitative research should examine service coordination in other programs and contexts that were not included in our study, such as tribal communities. Second, this study did not include the perspectives of other service providers within the maternal and early childhood system of care. Because service coordination inherently involves multiple individuals across service sectors, future research should incorporate these perspectives. Finally, this study focused on high performing programs; perspectives from programs that struggle with coordination are important and should be included in future research.

## Conclusions and Future Directions

Findings from this study reiterate the crucial role that home visiting programs play in connecting families with services and navigating complex systems of care and highlight the importance of increasing the efficiency of such processes (Goldberg et al., 2018; Minkovitz et al., 2016). The results of this multiple-case study of high-performing home visiting programs suggest action steps for states, local communities, and organizations to consider to facilitate service coordination. State and local leaders should support early childhood coalitions that include home visiting programs. Coalitions should also include representatives from organizations that provide mental and behavioral health services and that help families meet concrete needs, such as for housing. These coalitions require funding and infrastructure to assure implementation and sustainability. Several initiatives currently support such coalitions. For example, HRSA’s Early Childhood Comprehensive Systems Health Integration Prenatal-to-Three (ECCS) program awarded grants in 20 states, the Administration for Children and Families’ Preschool Development Grant Birth through Five awarded initial grants to six states and renewal grants to 23 states, and the Pritzker Children’s Initiative supports coalitions in 20 states and 11 communities. Coalitions are uniquely poised to identify strategies to promote service coordination. This study’s identification of facilitators and barriers to service coordination is an essential first step.

To our knowledge, this is the first qualitative inquiry into service coordination that analyzes data in the context of the Measurement Framework for Coordination, which aims to promote intentionality and use of evidence to strengthen coordination (West et al., 2018b). Previous service coordination research using quantitative methods has focused on developing indicators, refining program policies, and strengthening data systems (Rosinsky et al., 2019; West et al., 2018a). In this study, which relied on the perspectives of HVs and the families they serve, respondents highlighted the importance of “soft skills,” such as well-connected and dedicated staff, a workplace culture of teamwork, and building trusting relationships over time. These factors have not been the focus of prior work and may be difficult to measure quantitatively. Future research should recognize that these factors are integral to the practice of home visiting service coordination and continue to centralize the perspectives of frontline home visiting staff and families. Partnerships across diverse stakeholder groups are critical for generating research results that translate into practice and policy (Supplee & Duggan, 2019).

## Supplementary Information

B﻿elow is the link to the electronic supplementary material.

Supplementary file1 (PDF 33 KB)

## Data Availability

This study's data are not publicly available to protect the privacy of research participants.
