# Supplementary material for: Service Coordination in Early Childhood Home Visiting: a Multiple-Case Study
Source: Prev Sci. 2023 Jun 27;24(6):1225–38. doi: 10.1007/s11121-023-01558-6 (PMC10423702; doi:10.1007/s11121-023-01558-6)
Supplement: Supplementary file 1 — Supplementary file1 (PDF 33 KB) [file 11121_2023_1558_MOESM1_ESM.pdf]

Supplemental Content. Measurement Framework for Service Coordination in Home Visiting

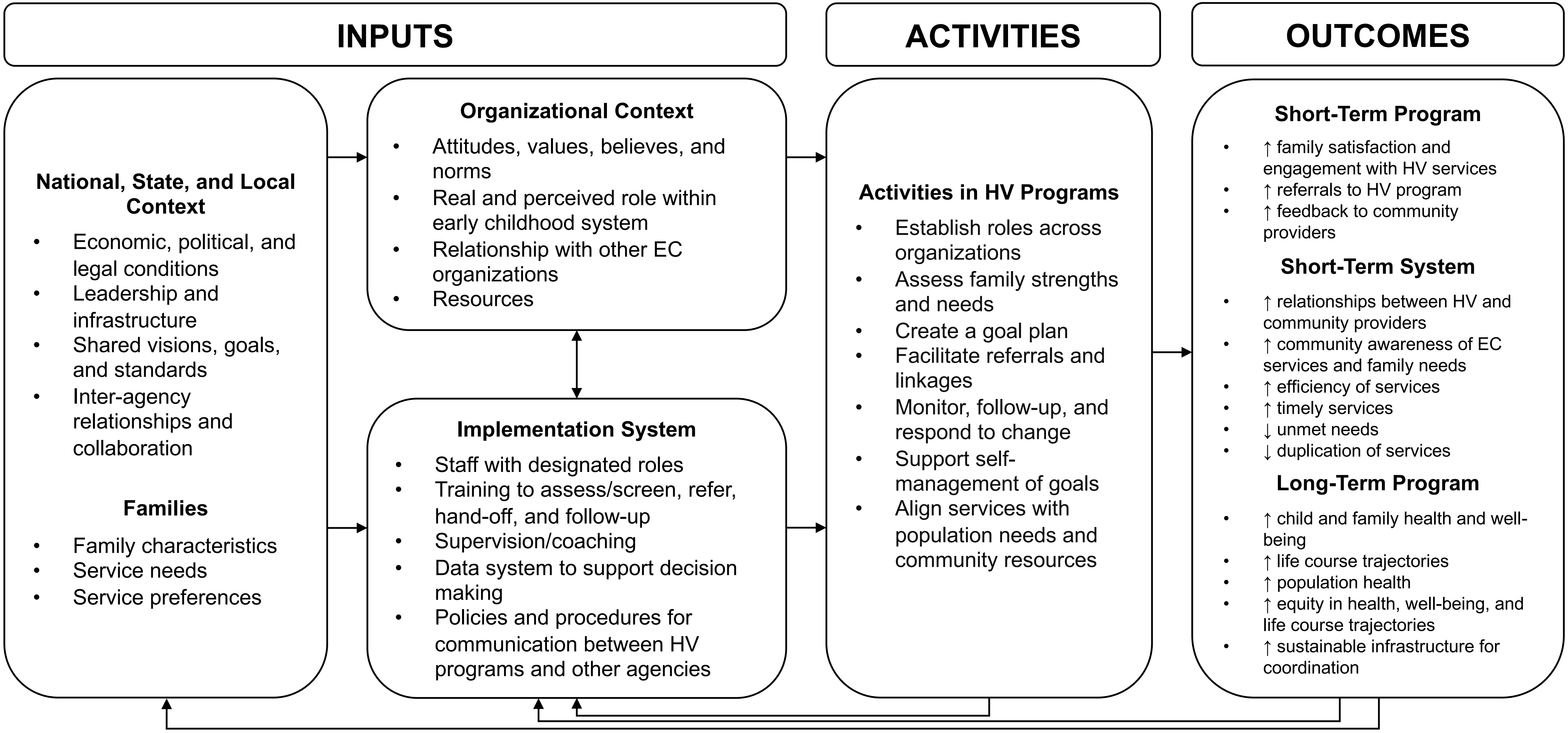

[1]Adapted from https://www.hvresearch.org/service-coordination-toolkit/jhu-framework/
